# Suitability of Mycorrhiza-Defective Rice and Its Progenitor for Studies on the Control of Nitrogen Loss in Paddy Fields via Arbuscular Mycorrhiza

**DOI:** 10.3389/fmicb.2020.00186

**Published:** 2020-02-07

**Authors:** Shujuan Zhang, Zhaoyang You, Xinyue Guo, Wenfei Yun, Yu Xia, Matthias C. Rillig

**Affiliations:** ^1^College of Urban Construction, Nanjing Tech University, Nanjing, China; ^2^Institut für Biologie, Plant Ecology, Freie Universität Berlin, Berlin, Germany; ^3^Berlin-Brandenburg Institute of Advanced Biodiversity Research, Berlin, Germany

**Keywords:** mycorrhiza-defective rice, nitrogen loss, runoff, leachate, N_2_O, paddy fields

## Abstract

Employing mycorrhiza-defective mutants and their progenitors does not require inoculation or elimination of the resident microbial community in the experimental study of mycorrhizal soil ecology. We aimed to examine the suitability of mycorrhiza-defective rice (non-mycorrhizal, *Oryza sativa* L., cv. Nipponbare) and its progenitor (mycorrhizal) to evaluate nitrogen (N) loss control from paddy fields via arbuscular mycorrhizal (AM) fungi. We grew the two rice lines in soils with the full community of AM fungi and investigated root AM colonization. In the absence of AM fungi, we estimated rice N content, soil N concentration and microbial community on the basis of phospholipid fatty acids; we also quantified N loss via NH_3_ volatilization, N_2_O emission, runoff and leaching. In the presence of AM fungi, we did not find any evidence of AM colonization for non-mycorrhizal rice while mycorrhizal rice was colonized and percentage of root colonization was 17–24%. In the absence of AM fungi, the two rice lines had similar N content, soil N concentration and microbial community. Importantly, there was no significant difference in N loss via all the four pathways between mycorrhizal and non-mycorrhizal systems. This mycorrhizal/non-mycorrhizal rice pair is suitable for further research on the role of AM fungi in the control of soil N loss in paddy fields.

## Introduction

In rice production, nitrogen (N) is one of the main limiting nutrients, requiring N fertilizer application to enhance rice productivity. Currently, the amount of N fertilizers applied is intensive and excessive in China. Specifically, China produces 19% of the world’s food supply while using an amount of chemical fertilizer equivalent to 30% of the world’s annual food consumption during the last decade (West et al., 2014). However, less than 35% of N fertilizers applied can be taken up by rice in the current growing season (Cao and Yin, 2015); the rest of N is transported into surrounding water systems via runoff and leaching and also lost via N_2_O emission and NH_3_ volatilization. According to a study conducted by Chen et al. (2014), approximately 25% of N was lost from rice paddy field via runoff; 14% of N via leaching, 59% via NH_3_ volatilization and 2% via N_2_O emission. Importantly, Wang et al. (2018) reported that NH_3_ volatilization from paddy fields was underestimated by 33% in China. Taken together, N loss does not only lead to degradation of water systems, but it also contributes to greenhouse gas emission directly via N_2_O emission and indirectly via NH_3_ volatilization (Lam et al., 2017). Therefore, it is crucial to reduce N loss from paddy fields not only to mediate water degradation but also to cope with climate change.

Arbuscular mycorrhizal (AM) fungi, forming symbioses with more than 80% of terrestrial plants (Smith and Read, 2008), show great potential to reduce N loss from soil (Cavagnaro et al., 2015). They can cut down N loss via several pathways, such as runoff, leaching and N_2_O emission. In the runoff pathway, our earlier field study conducted in Northeast China suggested that inoculation with AM fungi reduced N loss via runoff, regardless of fertilizer levels (Zhang et al., 2016). For the leaching pathway, researchers reported that AM fungi were able to reduce N leaching from experimental grasslands (Köhl and van der Heijden, 2016). Aside from runoff and leaching, AM fungi were reported to remarkably reduce N_2_O emission from paddy field soil (Zhang et al., 2015b). Importantly, two N loss pathways (i.e., leaching and N_2_O emission) are cut down by AM fungi in a single experiment, without an apparent tradeoff between these two pathways (Bender et al., 2015). Therefore, AM fungi show great potential for application in the reduction of N loss from paddy fields.

Arbuscular mycorrhizal fungi are ubiquitous in soils, requiring experimental approaches to creating an AM fungal treatment and control. Several types of possible interventions for AM fungi represent a gradient of realism and degree of experimental control. One of them is intercropping/rotating with host (as treatment) and non-host plants or a fallow stage (as control) (Higo et al., 2010; Koide and Peoples, 2012). Other studies have used reduced/no tillage to create an AM fungal treatment, paired with conventional tillage for a corresponding control, where the extraradical hyphae are reduced (de Pontes et al., 2017). The disadvantage of these two intervention types, while capturing realistic scenarios, is that intercropping, crop rotation and tillage treatments might result in other changes, such as soil nutrient availability and physical structure, which leads to difficulties in attributing any effects to AM fungi. Researchers also establish their control and treatment by applying soil fumigation (An et al., 1993) or fungicides (Landry et al., 2008). These interventions, however, cause non-target effects on soil microorganisms other than AM fungi. It is clear that these experimental treatments have disadvantages for studies on N loss control from paddy fields with AM fungi.

Aside from the above intervention types, there are other strategies offering the experimental control. For example, compartments with and without access to AM hyphae are designed to investigated the role of AM fungi in N cycling (Hodge et al., 2001; Storer et al., 2017). This experimental system is appropriate to study upland ecosystems, but not for wetlands (such as paddy fields) because of possible water flow and mass flow between compartments. Inoculation after soil sterilization is another widely used intervention type (Bitterlich et al., 2018); however, this is limited to typically a few cultured AM fungal isolates, and this excludes capturing effects of the full community of indigenous AM fungi. Additionally, researchers also designed static/rotated meshed core arrays which permit the creation of mycorrhizal hypha-free soil compartments (Johnson et al., 2001). This method interrupts the growth of AM fungi in control, that is, it is not disturbance free. Therefore, it is still urgent to explore other intervention types to create AM fungal treatment and control.

Compared with all the above intervention types, using mycorrhiza-defective mutants (non-mycorrhizal) and their progenitor (mycorrhizal) are particularly advantageous to study N loss control via AM fungi (Watts-Williams and Cavagnaro, 2015). This intervention type is non-invasive, disturbance-free and captures the entire community of AM fungi. Only few pairs of mutants and their progenitors are available, such as the well-known tomato mutant *rmc* and its wild type *76R* (Barker et al., 1998; Gao et al., 2004; Cavagnaro et al., 2012; Watts-Williams and Cavagnaro, 2014; Bowles et al., 2016) and FatM mutant lines and their wild type of *Lotus japonicas* (Brands et al., 2018). However, for these non-mycorrhizal/mycorrhizal pairs, data are not yet available that would permit their use in asking questions in mycorrhizal soil ecology (Rillig et al., 2008). According to Rillig et al. (2008), two conditions are necessary to make such a non-mycorrhizal/mycorrhizal pair useful: (i) in the presence of an entire AM fungi community derived from a complex inoculum source (roots, hyphae, and spores) with the full soil microbiota background, the mycorrhizal genotype (s) will not and the WT genotype will become colonized by AM fungi, and (ii) grown in the absence of AM fungi, they will exhibit similar growth parameters and will give rise to similar soil microbial communities.

The demand of such non-mycorrhizal/mycorrhizal pairs in rice is increasing to address questions related to the role of AM fungi in N loss control. Therefore, we here evaluated a non-mycorrhizal/mycorrhizal pair of rice for its suitability in addressing questions about N loss control. On the basis of the two conditions mentioned above (Rillig et al., 2008), we established our own evaluation criteria for this purpose, including:

Criterion (i): With a native AM fungal community and its soil microbiota background, the non-mycorrhizal rice will show resistance to AM fungi;
Criterion (ii): In the absence of AM fungi, mycorrhizal and non-mycorrhizal plants will have similar N content and comparable effects on soil N concentration;
Criterion (iii): In the absence of AM fungi, there is no difference in soil microbial communities between mycorrhizal and non-mycorrhizal rice;
Criterion (iv): In the absence of AM fungi, mycorrhizal and non-mycorrhizal systems will have the same pattern of N loss, via runoff, leaching, N_2_O emission and NH_3_ volatilization, thus indicating the absence of non-target effects on the processes of interest here.

## Materials and Methods

### Plant Material and Soil

Two lines of rice (*Oryza sativa*, L. ssp. Japonica cv. Nipponbare) were used in this experiment. One is mycorrhiza-defective (here referred to as ‘non-mycorrhizal’) because its *CERK1* gene required for mycorrhizal penetration and colonization, is blocked with RNA interference technology (Zhang et al., 2015a). Compared with this non-mycorrhizal line, its progenitor was able to form symbiosis with AM fungi, here referred to as ‘mycorrhizal.’

Rice seeds of the two rice lines were surface sterilized in 75% ethanol for 10 min, then thoroughly rinsed with sterile reverse osmosis water to remove any residual ethanol. Then, these seeds were kept in an incubator for 72 h at 28°C in the dark for germination. After this, they were ready to be sown.

The soil used in our study was collected from a paddy field in Changshu Agro-ecological Experimental Station, Chinese Academy of Sciences, Changshu City, Jiangsu Province, China (31°32′93′′ N, 120°41′88′′ E). We gathered 10 random soil samples (with a depth of 20 cm) from this area. After pooling and homogenization, these samples were used for soil tests. The soil texture is silt clay loam, with 13% sand, 55% silt, and 32% clay. The soil in this region contained 2.8 g kg^–1^ total nitrogen, 26.6 g kg^–1^ organic C, 0.9 g kg^–1^ total P and had a pH of 7.0 (soil: MiliQ water = 1:2.5) at the beginning of the experiment. AM fungi were abundant at this site, with the percentage of root colonization varying from 15 to 73% during the rice growing season (Zhang et al., 2019). The soil was air dried and passed through a 2 mm round-hole sieve.

### Experiment Design

We conducted three experiments; we ran Experiment 1 and Experiment 2 to test Criterion (i): whether the non-mycorrhizal rice line show resistance to a native AM fungi community of a typical paddy field. Experiment 3 was conducted to test the remaining criteria in the absence of AM fungi, that is, whether the two rice lines have similar effects on N cycling factors.

In Experiment 1, the two rice lines were grown separately with the original soil collected without any sterilization. There were four biological replicates for each rice line. In this experiment all the propagule types, e.g., spores, hyphae and colonized root fragments, were present (Zhang et al., 2019). In this experiment, the rice was grown in soil medium without mixed with sand.

We ran Experiment 2 in the same way of Experiment 1. The only difference is that in Experiment 2, we used a soil–sand mixture to grow rice while in Experiment 1 we only used soil. Our pretest revealed that if we only use soil (without sand) to pack the experimental microcosms (see below), we could not get enough samples for the following tests even after leaching for 24 h. Therefore, soil was mixed with sand at a ratio of 40:60 (w:w) and then used to pack the microcosms in Experiment 2 and Experiment 3. The soil/sand mixture was also utilized to facilitate watering and root extraction. Our pretest showed that water filtration was quite slow when we watered the plants and it was also hard to separate roots from soil particles.

In Experiment 3, we grew the two rice lines separately with autoclaved soil. There were four biological replicates for each rice line. In this experiment, soil was autoclaved twice at 121°C for 2 h to eliminate any AM fungi. Then, it was mixed with sand at a ratio of 40:60 (w:w) (the same as in Experiment 2). To stimulate the effect of water flow on a soil surface in paddy fields, soil (not soil/sand mixture) was used for the top layer (0–5 cm). In order to reintroduce other soil microbiota after sterilization, 10 mL of a filtered wash of the non-sterilized soil was added to each unit. The filtered wash was obtained by passing soil solutions through a 10 μm nylon filter from 5 g original non-autoclaved soil (this method is sufficient to exclude any AM fungal propagules) (Rillig et al., 2008).

### Experimental Microcosms

**FIGURE 1:**
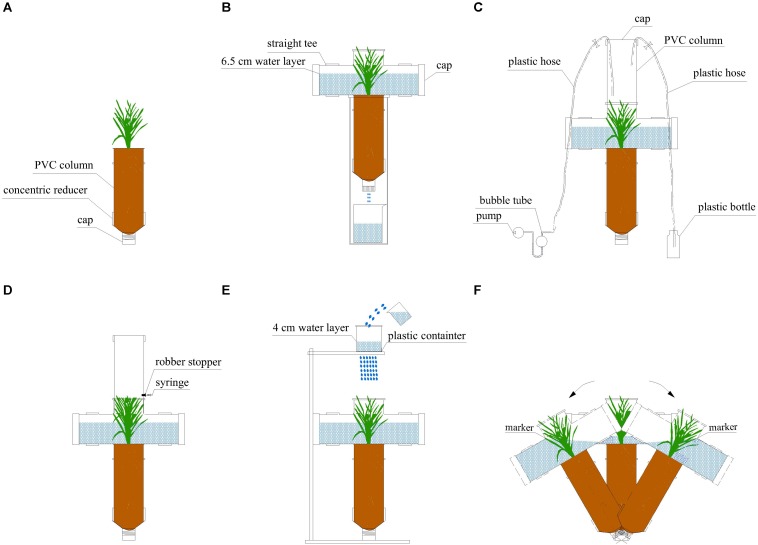
Schematic of experimental microcosms used in our experiment. **(A)** A plant growth unit with rice growing; **(B)** a plant growth unit with a leachate collecting device; **(C)** NH_3_ sampling; **(D)** N_2_O sampling; **(E)** a plant growth unit with an artificial rainfall generator; **(F)** rocking of the plant growth unit before runoff water collection.

The microcosm had a plant growth unit for rice growth and devices for sampling. The plant growth unit had two parts (Figure 1A). A PVC column had a diameter of 110 mm and a depth of 300 mm and it was for rice growth. On the bottom of this column, there was a concentric reducer (diameter: 110 and 50 mm) with a cap to collect leachate. Two layers of gauze were placed inside the concentric reducer to avoid any contamination.

Afterward, the plant growth units were assembled as follows: two layers of gauze were packed firstly, then 0.2 kg of washed and dried quartz sand (diameter: 2–4 mm), followed by soil–sand mixture [autoclaved soil: sand = 40:60 (w:w)]. The units were not fully filled, with the top 5 cm layer unfilled. Then, six germinated seeds were grown and covered with only soil (non-autoclaved soil in Experiment 1 and Experiment 2 but autoclaved soil in Experiment 3).

All plants were cultivated in a light growth chamber with 12 h light (5500 Lux), at 28°C during day time and 24°C at night. The plants were irrigated with 50 mL of tap water each day. After 7 days, four similar seedlings were kept for each plant growth unit. Therefore, there were 16 seedlings (four plants in each replicate; four replicates) in total for each rice line in the three experiments. During this period, the plants were not fertilized.

### Fertilization and Sampling

In Experiment 1 and Experiment 2, after 4 weeks of growth plants were harvested and roots were collected for AM colonization measurement. In these two experiments, we did not fertilize the plants.

In Experiment 3, after 4 weeks of growth we conducted two rounds of sampling, before and after fertilization, respectively. Then, we harvested the plants, with shoots and roots separated. Based on the common agricultural practice in the southeast and northwest of China, rice seedlings had four leaves after 4 weeks growth since sowing. At this stage, they were ready for fertilization and flooding condition, that was, a water layer at a depth approximately 3–10 cm above the soil surface. In addition, this is the rainy season in these two regions so that runoff and leaching are very common during this period. Furthermore, our previous test indicated that the two rice lines showed difference in biomass and N content at this stage when they were inoculated with AM fungi (unpublished data). In accordance with the common agricultural practice and rainfall characteristics of the region where the paddy field soil was collected, we conducted the irrigation, fertilization and sampling in Experiment 3.

Before sampling, we took all the plant growth units out of the growth chamber and installed a straight tee (110 mm diameter) on the top of each unit. This straight tee was utilized to hold a water layer above soil surface 6.5 cm (Figure 1B) before sampling. This was to stimulate the real situation of rice growth at this stage. During sampling, this straight tee could support the devices for N_2_O, NH_3_ and runoff collection (Figures 1C–F).

After 24 h, we conducted the first round of sampling as follows, recorded as ‘before fertilization.’

NH_3_ sampling was carried out for 1 h with a dynamic chamber method (Figure 1C): we installed another PVC column (diameter: 110 mm; height: 30 cm; volume: 2.8 L) with a cap on the straight tee. We set the airflow rate of the pump at 1 L min^–1^ and used 10 mL sulfuric acid (0.005M) to absorb NH_3_ in the samples. A 1.5 L bottle placed two meters away (to avoid disturbance) was connected to supply ambient air (Wang et al., 2017).

We collected N_2_O samples with the static chamber method (Zhang et al., 2015b). We closed the chamber (made of a PVC column having a volume of 2.8 L) for 2 h and then collected samples with a 200-mL syringe (Figure 1D). After this, we removed the cap at the bottom of the concentric reducer and collected leachate with a beaker for 3 min (Figure 1B). The leaching flow rate was around 1.3 mm s^–1^. We have to highlight that when leachate was collected a water layer was kept in the microcosms so that there was no delay from the start of irrigation to the end of water sampling. Then, 500 mL of recovered water was gently added to keep the water layer at 6.5 cm depth.

We applied artificial rainfall at 100 mm min^–1^ for 10 min to sample runoff water (Figure 1E). This was achieved with a simplified artificial rainfall generator. This rainfall generator was a plastic container with uniform holes set up 100 cm above the straight tee. When water in the plastic container was kept at 4 cm depth, the rainfall intensity was maintained at 100 mm min^–1^. During this rainfall event, the water did not infiltrate because there was already a water layer before this rainfall. In addition, the water did not run over the edges of the containers as the straight tee has enough volume for the rainwater.

Following this rainfall event, we rocked the entire system from side to side in one direction (no rotation) to stimulate the impact of water flow on the soil surface during runoff (Figure 1C). In order to make this rocking reproducible, we marked inside the straight tee at 8.5 cm above soil surface and the rocking ended when the water reached this marker. During the whole rocking process, no slowing down and acceleration occurred and the soil surface was covered with water at all times. We conducted this rocking 20 times, which roughly took 2 min.

Runoff water samples were subsequently collected immediately. The unit was kept leaning to one side, then we pour out 500 mL of water into a beaker from the straight tee. These water samples were taken as runoff samples. After homogenization, each water sample was split into two: one passed through a 0.45 μm filter membrane to measure dissolved N (DN), ${\text{NO}}_{3}^{-}$-N, ${\text{NO}}_{2}^{-}$-N, ${\text{NH}}_{4}^{+}$-N concentrations; the other one was used to measure total N (TN) concentration without filtration. After runoff water was sampled, we added water gently to the microcosms to keep the original water layer (6.5 cm, Figure 1B).

After this first round of sampling, the plants were fertilized directly with 300 kg N ha^–1^ with urea, 180 kg P_2_O_5_ ha^–1^ with superphosphate and 60 kg K_2_O ha^–1^ with potassium chloride. These fertilizers were directly applied into the water layer above the soil surface.

One day after fertilization, we conducted the second round of sampling as above, recorded as ‘after fertilization.’ The 24 h interval might be not long enough compared with the real situation. We made this decision based on Bender et al. (2015). In this paper, leachate was collected 24 h after fertilization and importantly, AM fungi played considerable role in reducing nutrient concentrations of the leachate.

After all the above sampling was completed (2 days after fertilization), the shoots and roots of the two rice lines were harvested and washed. Each root was separated equally into two parts: one for root colonization measurement while the other, together with shoots, was dried in an oven (75°C, 72 h) for biomass and N concentration measurement.

As the top layer (0–5 cm) of the plant growth medium contained the inoculum and most of the rice roots, samples were taken from this layer and air-dried to measure plant available N concentration.

To analyze the plant effects on the soil microbial community in the absence of AM fungi, we used phospholipid fatty acids (PLFAs) analysis. For this purpose, 50 g fresh soil was collected from each unit after plant harvesting and immediately frozen after mixing.

### Sample Measurement

The percentage of root colonization was estimated after acid fuchsin (Aladdin Industrial Corporation, Shanghai, China) staining with a microscope (Eclipse E 200; Nikon Instruments, Beijing, China) using the grid line intersects method (Giovannetti and Mosse, 2010). N concentrations of plant shoot and root were determined with an Elemental analyzer (vario EL III, Germany). The plant available N concentrations of soil were tested with the Alkali hydrolysis diffusion method [Forestry industry standard of China (LY/T 1229-1999)].

We extracted PLFAs from 2.0 g soil samples in a buffered chloroform/methanol solution. These PLFAs were separated from other lipids with silicic acid chromatography, derivatized to fatty acid methyl esters and then quantified with gas chromatography (Ramsey et al., 2005). In total, we obtained approximately 41 PLFA markers in our samples. The microbial community richness was considered as the total number of fatty acid peaks identified in each chromatogram (Ramsey et al., 2005). The total PLFA content (nmol g^–1^ dry soil) was reported as a measure of microbial biomass (Frostegård et al., 1991). For all of the PLFA analysis we only had three replicates, as the fourth one was not properly stored.

We report changes in the abundance of specific populations of microbes by using the abundance of specific fatty acids as signature lipids. Specifically, the straight chain fatty acids (14:0, 15:0, 16:0, 18:0, 20:0, 22:0, and 24:0) were regarded as general bacteria (Moon et al., 2016); the Gram-negative bacteria were indicated with monoenoic and cyclic fatty acids (18:1ω9c, 16:1ω9c, cy17:0, and cy19:0) (Zelles et al., 1992); the Gram-positive bacteria were traced with several branched fatty acids (a15:0, i16:0, and i17:0) (Zelles et al., 1992); 10me16:0, 10me17:0, and 10me18:0 served as the proxies of Actinomycetes (Ramsey et al., 2005); the fatty acid 18:2ω6c was used as the biomarker of fungi (Frostegård and Tunlid, 1993).

N_2_O fluxes were analyzed with a chromatograph (Agilent 7890), and NH_3_ fluxes were analyzed by sodium hypochlorite-salicylic acid spectrophotometry [National Environmental Protection Standard of China (HJ 534-2009)].

For runoff water and leachate, total nitrogen (TN), dissolved nitrogen (DN), particulate nitrogen (PN), ammonia nitrogen (${\text{NH}}_{4}^{+}$-N), nitrate nitrogen (${\text{NO}}_{3}^{-}$ -N), nitrite nitrogen (${\text{NO}}_{2}^{-}$ -N) and dissolved organic nitrogen (DON) concentrations were measured. TN concentrations were measured with the ultraviolet spectrophotometry method with potassium peroxydisulfate. DN concentrations were also assessed with this method but with filtrate of a 0.45 μm filter membrane while the difference between TN and DN were considered as PN. ${\text{NH}}_{4}^{+}$-N concentrations were measured with Nesster’s reagent by colorimetry. ${\text{NO}}_{3}^{-}$ -N concentrations were measured based on spectrophotometry with phenoldisulfonic acid while ${\text{NO}}_{2}^{-}$-N concentrations were estimated with *N*-(1-Naphthalene)-Diaminoethane colorimetry. All of these methods are standard methods in Quality Standard of Surface Water Environment of China (GB3838-2002). DON concentrations were calculated by subtracting the concentrations of ${\text{NH}}_{4}^{+}$ -N, ${\text{NO}}_{3}^{-}$ -N and ${\text{NO}}_{2}^{-}$-N from the DN concentrations.

### Calculations

NH_3_ fluxes were calculated based on equation (1) (Cao et al., 2013) and cumulative N loss via NH_3_ volatilization was the sum of NH_3_ volatilization fluxes on sampling days.

(1) $$F_{N\,H\,3}\,\left(m\,g\,m^{-2}\,h^{-1}\right)=\frac{A}{S\times H}$$

where A is the total ammonia content in the sample; S is the effective area of NH_3_ sampling; H is the time of sampling.

N_2_O fluxes were calculated based on equation (2) (Wang et al., 2014) and cumulative N loss via N_2_O emission was the sum of N_2_O emission fluxes on sampling days.

(2) $$F_{N\,20}\,\left(m\,g\,m^{-2}\,h^{-1}\right)=\rho \times \frac{V}{A}\times \frac{d\,C}{d\,t}\times \frac{273}{273+T}$$

where ρ is the N_2_O density under standard conditions; V is the effective volume of N_2_O sampling; A is the effective area of N_2_O sampling; dC/dt is the change rate of N_2_O concentration (dt is 2 h; dC is the difference in N_2_O concentration between when we closed the static chamber and 2 h later; ambient N_2_O concentration is the baseline); T is the air temperature.

### Statistical Analysis

The normality and homogeneity of variance for all data were tested. *t*-Tests were carried out to distinguish the effects of rice lines and fertilization on the predictors. Statistical analyses were performed with SPSS 20.0 (SPSS, Inc., Chicago, IL, United States) and figures were created with Origin 8.0 (OriginLab, Northampton, MA, United States).

To quantify community structural differences, we carried out principal component analysis based on the 30 most abundant PLFAs (mole ratio of each PLFA to the total PLFAs) present in each sample (Bååth and Tunlid, 1998). This principal component analysis was performed with individual PLFA values expressed as a proportion of the total PLFAs in a sample to remove the effect of biomass differences on the analysis (Ramsey et al., 2005).

## Results

### Root Colonization by AM Fungi

In the soil with a native AM fungi community background, the percentage of root colonization of mycorrhizal rice was 17 ± 2% in Experiment 1 and 23 ± 4% in Experiment 2. We did not detect any evidence of AM colonization in non-mycorrhizal plant roots in these two experiments. In the absence of AM fungi (Experiment 3), no AM colonization was observed in any rice line.

### Plant N Content and Soil N Concentration

**FIGURE 2:**
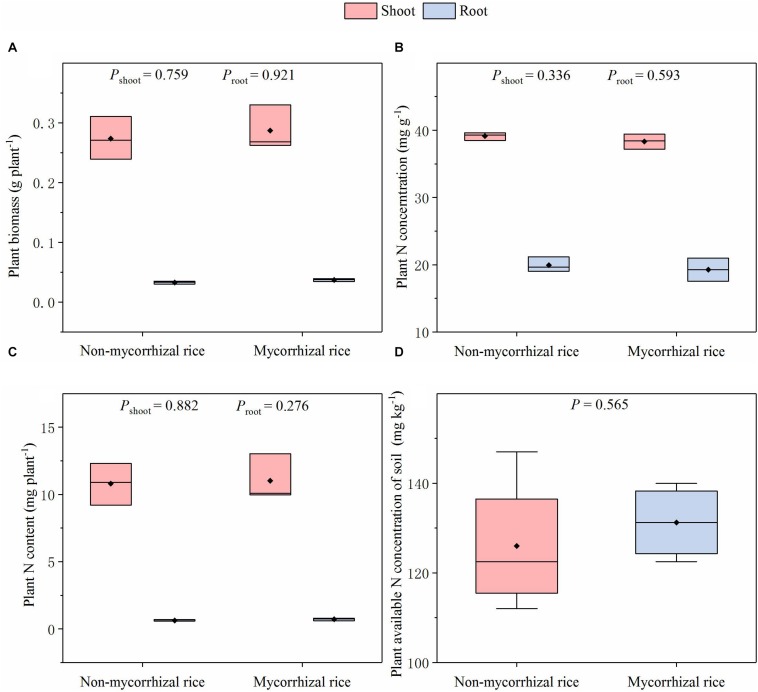
Biomass, N concentration, N content of rice and plant available N concentration of soil in the AM fungi absent situation (Experiment 3). *P*: significance of the difference in parameters between the two rice lines; diamonds in the boxplots represent the mean values; there are four replicates for each treatment. **(A)** Plant biomass; **(B)** Plant N concentration; **(C)** Plant N content; **(D)** Plant available N concentration of soil.

In the absence of AM fungi (Experiment 3), there was no difference in biomass for shoot and root between the two rice lines; the plant N concentrations were also similar; there was no difference in the plant N content between mycorrhizal and non-mycorrhizal rice lines (Figure 2). In addition, we did not observe differences in the plant available N concentration of soil (Figure 2).

### Soil Microbial Community

**FIGURE 3:**
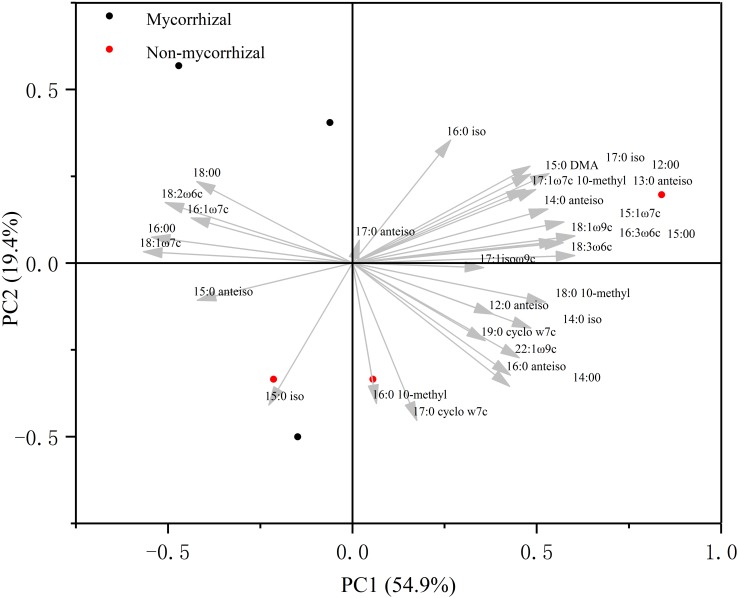
Principal component analysis ordination of microbial community composition (%) in Experiment 3. This analysis was conducted based on the most abundant 30 individual phospholipid fatty acid (PLFA) biomarkers.

In the absence of AM fungi (Experiment 3), we compared microbial communities of soil growing the two rice lines. In the principal component analysis, the first two important components explained 74% of the variance in the relative abundance of the individual fatty acids (Figure 3). Neither PC1 nor PC2 separated the microbial community structure with the mycorrhizal rice from that with the non-mycorrhizal rice.

The soil microbial biomass and PLFA richness, are represented with the sum total abundance of PLFAs and total number of fatty acid peaks identified in each chromatogram, respectively. Importantly, there was no difference in these two parameters between mycorrhizal and non-mycorrhizal systems (Table 1). The abundance of general bacteria, Gram positive and negative bacteria, actinomycetes and fungi were also similar between these two systems (Table 1).

**TABLE 1:** Characteristics of soil microbial communities with mycorrhizal and non-mycorrhizal rice lines in Experiment 3.

**Community indicators**	**Mycorrhizal**	**Non-mycorrhizal**	***P***
Microbial species richness (peaks)	41.33 ± 1.70	40.67 ± 1.89	0.73
Microbial biomass (nmol PLFA g^–1^ soil)	12.76 ± 0.99	11.97 ± 0.70	0.42
General bacteria (nmol PLFA g^–1^ soil)	3.34 ± 0.42	2.87 ± 0.45	0.35
Gram positive bacteria (nmol PLFA g^–1^ soil)	1.26 ± 0.12	1.15 ± 0.08	0.35
Gram negative bacteria (nmol PLFA g^–1^ soil)	1.47 ± 0.16	1.29 ± 0.14	0.28
Actinomycetes (nmol PLFAs g^–1^ soil)	0.66 ± 0.14	0.50 ± 0.13	0.29
Fungi (nmol PLFA g^–1^ soil)	0.39 ± 0.03	0.41 ± 0.04	0.76

*Values are shown as means ± SD (*n* = 3).*

### Fluxes of NH_3_ and N_2_O

**FIGURE 4:**
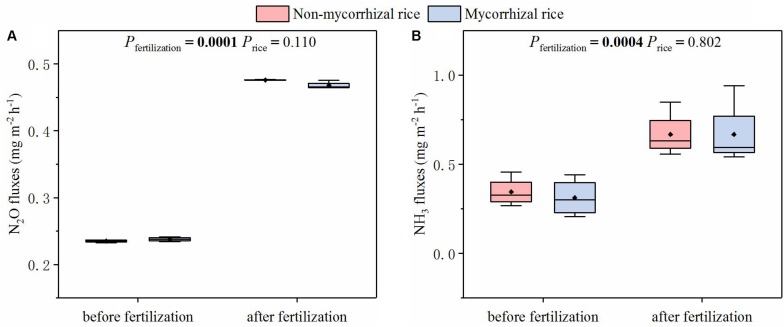
Emission fluxes of N_2_O **(A)** and NH_3_ **(B)** from systems with mycorrhizal and non-mycorrhizal rice in the absence of AM fungi (Experiment 3). *P*_*fertilization*_: the difference in fluxes between before and after fertilization; *P*_*rice*_: the difference in fluxes between mycorrhizal and non-mycorrhizal rice lines; diamonds in the boxplots represent the mean values; there are four replicates for each treatment.

With the two rice lines growing in the absence of AM fungi (Experiment 3), we measured the fluxes of NH_3_ and N_2_O before and after fertilization. There was no significant difference in the fluxes of NH_3_ and N_2_O between mycorrhizal and non-mycorrhizal systems, although both NH_3_ and N_2_O fluxes increased after fertilization (Figure 4).

### N Concentrations of Runoff Water and Leachate

**FIGURE 5:**
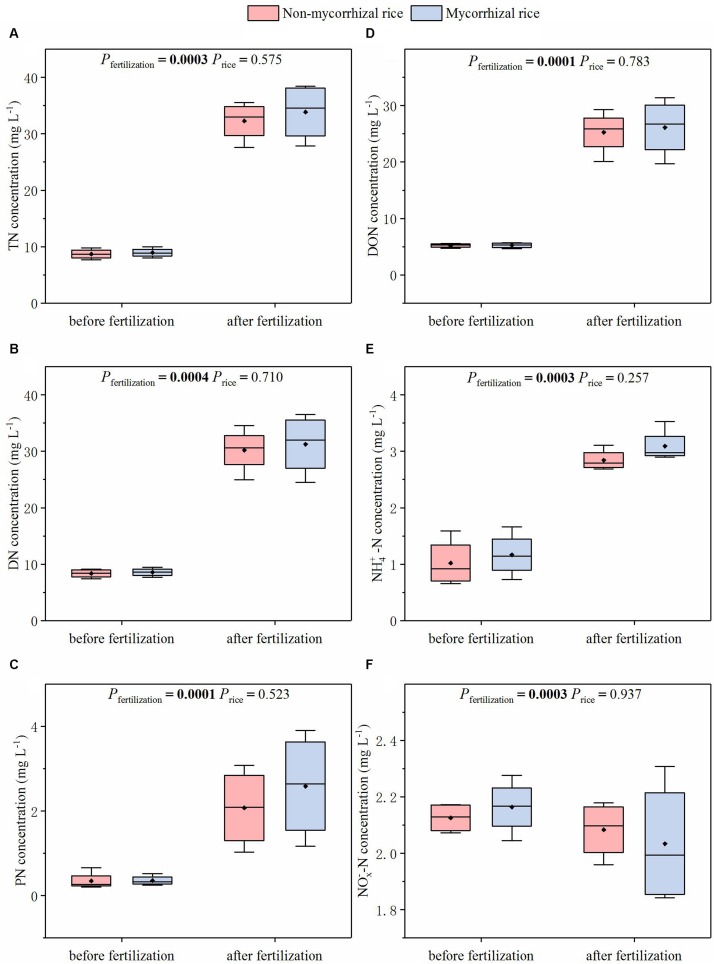
N concentrations of runoff water from systems with mycorrhizal and non-mycorrhizal rice in the absence of AM fungi (Experiment 3). TN: total nitrogen; DN: dissolved nitrogen; PN: particle nitrogen; ${\text{NO}}_{\text{X}}^{\text{-}}$-N: sum of ${\text{NO}}_{3}^{\text{-}}$ -N and ${\text{NO}}_{2}^{\text{-}}$-N; *P*_*fertilization*_: significance of the differences in N concentrations between before and after fertilization; *P*_*rice*_: significance of the differences in N concentration between the two rice lines; diamonds in the boxplots represent the mean values; there are four replicates for each treatment. **(A)** TN concentration; **(B)** DN concentration; **(C)** PN concentration; **(D)** DON concentration; **(E)** NH${}_{4}^{+}$-N concentration; **(F)** NO${}_{X}^{-}$-N concentration.

In the absence of AM fungi (Experiment 3), we monitored N concentrations in runoff water after the rainfall event. For all of the N forms, no difference was observed in N concentrations of runoff water between mycorrhizal and non-mycorrhizal systems (Figure 5). The fertilization increased concentrations of all these N forms (except ${\text{NO}}_{3}^{-}$-N, Figure 5).

**FIGURE 6:**
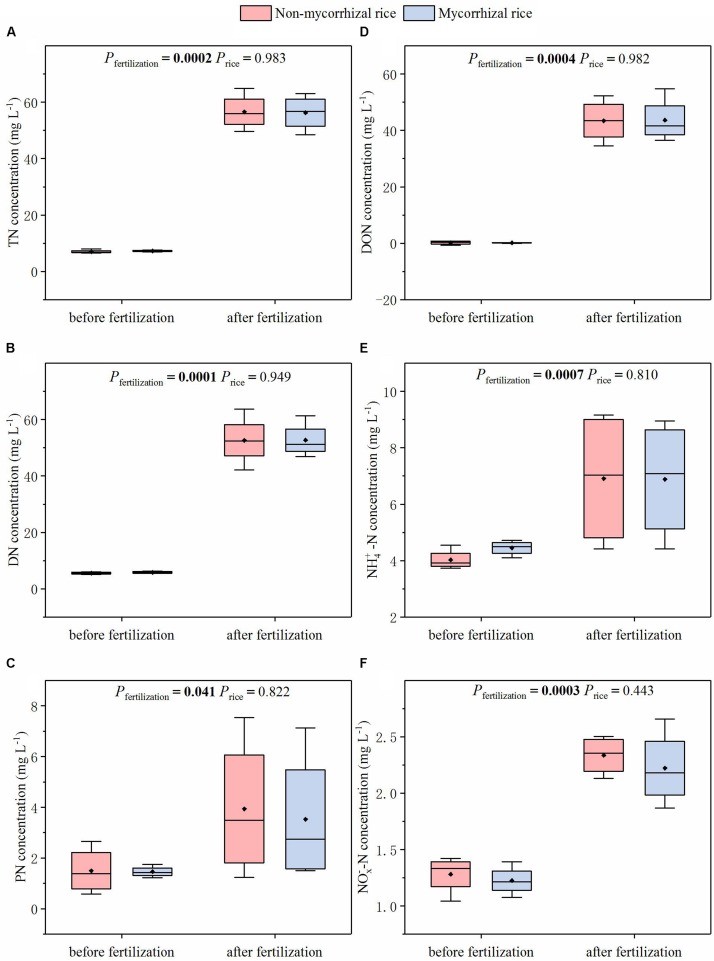
N concentrations of leachate from systems with mycorrhizal and non-mycorrhizal rice in the absence of AM fungi (Experiment 3). TN: total nitrogen; DN: dissolved nitrogen; PN: particle nitrogen; ^NO_X^–^ -N: sum of ${\text{NO}}_{3}^{-}$-N and ${\text{NO}}_{2}^{-}$ -N; *P*_*fertilization*_: significance of the differences in N concentrations between before and after fertilization; *P*_*rice*_: significance of the differences in N concentration between the two rice lines before and after fertilization, respectively; diamonds in the boxplots represent the mean values; there are four replicates for each treatment. **(A)** TN concentration; **(B)** DN concentration; **(C)** PN concentration; **(D)** DON concentration; **(E)** NH${}_{4}^{+}$-N concentration; **(F)** NO${}_{X}^{-}$-N concentration.

Similar to the runoff water, there was no difference in N concentrations of leachate between mycorrhizal and non-mycorrhizal systems (Figure 6). We also determined that the fertilization increased all N forms in leachate (Figure 6).

## Discussion

We aimed to assess a pair of mycorrhizal/non-mycorrhizal rice lines for their suitability in ecological studies on the role of AM fungi in decreasing N loss from paddy fields. We checked AM colonization of the two rice lines in soil with a full AM fungi community background. Additionally, in the absence of AM fungi, we investigated in detail plant N content, soil N concentration, microbial community and N loss from systems with mycorrhizal and non-mycorrhizal rice lines. Importantly, we validate that this mycorrhizal/non-mycorrhizal rice pair is suitable for studies on N loss control of paddy fields with AM fungi (Rillig et al., 2008).

Our study has relatively low replication (*n* = 4), which potentially led to each individual comparison having low statistical power. However, we also had quite low variability (for all the parameters, 80% of the coefficient of variation was lower than 0.20 and the mean value was 0.27), owing to the highly controlled conditions under which we carried out the experiments. In addition, we did not adjust critical *P*-values for the 38 comparisons made (i.e., an inflation of study-wide type I error rate is likely), which does work in favor of detecting responses if any were present.

### Meeting Criterion (i): Non-mycorrhizal Rice Showed Resistance to AM Colonization

In our Experiment 1 and Experiment 2, AM fungi provided to rice were from soil collected from a typical paddy field in Taihu Lake region, southeast of China. In this area, AM fungi were abundant, with the percentage of root colonization ranging from 15 to 73% and the spore density from 60 to 80 spores of 100 g soil (Zhang et al., 2019). With this soil and its native AM fungi and other soil microbiota present, the mycorrhizal rice line was colonized while its non-mycorrhizal counterpart was not in both Experiment 1 and Experiment 2. This revealed that the non-mycorrhizal rice had resistance to AM fungi, meeting Criterion (i). This resistance, the difference in AM colonization between this rice pair (17 and 23% in these two experiments), was smaller than 46% of another pot study (Zhang et al., 2015a). In spite of this, it is completely acceptable to test the AM effects on N loss from paddy fields on the basis of our previous field experiments where the difference in AM colonization between treatment (12–20%) and control (3%) was also small (Zhang et al., 2016).

This varied resistance of non-mycorrhizal plants to AM fungi might be attributed to both AM fungi and soil microbiota (Gao et al., 2010). For AM fungi, in this previous study only a specific AM fungal isolate (*R. irregularis*; no other AM fungi species) was provided (Zhang et al., 2015a); while in our study a native AM fungi community from a typical paddy field was present. This lower AM colonization for mycorrhizal rice in our study might be related to the competition for carbon among AM fungi species (Liu et al., 2015). In terms of other soil microbiota, in this previous study (Zhang et al., 2015a) the rice plants were cultivated in a mixture of sand and perlite (without soil microbiota); in our experiment, the rice plants were grown with soil microbiota from a typical paddy field. These soil microbiota might inhibit the growth of AM fungi because of the antagonistic interaction between them (Leigh et al., 2011). This was supported by our unpublished study where we demonstrated: when the two rice lines were inoculated with the same AM fungal isolate, the non-mycorrhizal rice was not colonized while AM colonization of the mycorrhizal rice was 21%. The result from this unpublished study is closer to what we found in Experiments 1 and 2, and is much lower than what was found in the study by Zhang et al. (2015a).

Aside from rice, there are such mycorrhizal and non-mycorrhizal pairs in other plant species, including tomato (*Solanum lycopersicum*), maize (*Zea mays*) and petunia (*Petunia hybrida*), which was well-summarized in a review (Watts-Williams and Cavagnaro, 2015). For these plants, their non-mycorrhizal mutants did not show consistent resistance to AM fungi all the time (Watts-Williams and Cavagnaro, 2014). For example, in a field experiment conducted in California the mycorrhizal tomato had an AM colonization of 20% while that of its mutant was 3% (Cavagnaro et al., 2012). In another field experiment also carried out in California (very close to the above experimental site), this resistance was smaller: 12 and 2% for mycorrhizal and non-mycorrhizal plants, respectively. Therefore, the suitability of these pairs must be tested if they are considered a tool in new circumstances.

### Meeting Criterion (ii): In the Absence of AM Fungi, the Two Rice Lines Had Similar N Content and Soil N Concentration

To assess the potential of AM fungi in mitigating N loss from paddy fields, it is critical to use rice lines with similar N uptake capability; otherwise, it will be hard to distinguish the role of AM fungi from that of the rice plants. In our study, the N uptake capability was indicated with plant N content, obtained via multiplying the biomass by the N concentration. Therefore, both plant biomass and N concentration determined N uptake capability. Importantly, not all of the previously examined mycorrhiza-defective mutants had similar biomass of shoots and/or roots as their progenitors in the absence of AM fungi (Watts-Williams and Cavagnaro, 2014). For example, the mycorrhiza-defective tomato mutant M161 had larger roots than its mycorrhizal wild type in the absence of AM fungi (Rillig et al., 2008). In our study, the mycorrhizal and non-mycorrhizal rice had similar biomass in the absence of AM fungi, which is in agreement with the tomato mutant *rmc* and its wild type (Cavagnaro et al., 2004). In this aspect, this rice pair supports criterion (ii).

For plant N content, in the presence of AM fungi, it is common for mycorrhizal plants to have higher N content than mutants (Cavagnaro et al., 2012; Bowles et al., 2016). In the absence of AM fungi whether mycorrhizal plants and their non-mycorrhizal mutants have similar N content, however, is not clear. The reason is likely that only few studies growing these plants in the absence of AM fungi have been conducted (Cavagnaro et al., 2004; Rillig et al., 2008). In our Experiment 3, we determined that there was no detectable difference in N concentrations for both shoot and root, and then comparable plant N content, that is, N uptake capability (Figure 2C). This indicated that for N uptake, the two rice lines are suitable for studies on the AM effects on N loss from paddy fields. We also found that there was no detectable difference in plant available N concentration of soil, which is important because soil is the direct source of N loss in paddy fields (Guo et al., 2004) (Figure 2D). Together with their similar N uptake capability, this rice pair had similar effect on soil N concentrations, meeting criterion (ii).

### Meeting Criterion (iii): In the Absence of AM Fungi, the Two Rice Lines Gave Rise to Similar Soil Microbial Communities

N loss is one of the outcomes of soil N cycling, an almost entirely microbially-driven cycle, and plant–microbe interactions may have a dramatic influence on the actual rates of N-cycling (Craine et al., 2007). Therefore, whether mycorrhizal and non-mycorrhizal rice lines had similar effects on the soil microbial community is an important criterion. Although the wild type tomato and its mutants are different in their root exudates (Sun et al., 2012), there was no difference in microbial community of soil growing the wild type tomato and its three mutants in the absence of AM fungi based on PLFAs (Rillig et al., 2008). In our study, we compared the two microbial communities by analyzing PLFAs. We used as many biomarkers as we could, including PLFA richness, microbial biomass, general bacteria, Gram-positive bacteria, Gram-negative bacteria, actinomycetes, and fungi. In spite of this, we did not observe any differences in these biomarkers between the two rice systems (Table 1). Additionally, a principal component analysis for PLFAs did not reveal any difference in community structure even though the two most important components we used accounted for 74% of variance (Figure 3). All these outcomes illustrated that this mycorrhizal/non-mycorrhizal rice pair met criterion (iii).

### Meeting Criterion (iv): In the Absence of AM Fungi, N Loss Was Similar for the Two Rice Lines

The AM effect on N loss from paddy fields is our research focus. The reason is that several studies reported that AM fungi played a role in reducing N loss from soil (Asghari and Cavagnaro, 2011, 2012; Cavagnaro et al., 2015). Importantly, this function of AM was confirmed with the mycorrhiza defective tomato mutant and its mycorrhizal wild type progenitor (Asghari and Cavagnaro, 2012). In order to avoid confounding the AM effects on N loss from paddy fields, we tested N loss from systems growing mycorrhizal or non-mycorrhizal rice lines. Because N loss from paddy fields occurred via runoff, leaching, N_2_O emission and NH_3_ volatilization (Chen et al., 2014), we compared fluxes of NH_3_ and N_2_O and N concentrations of runoff water and leachate between these two systems. In the absence of AM fungi, we determined that there was no significant difference in the fluxes of NH_3_ and N_2_O between the two rice systems (Figure 4). We also found that for runoff water and leachate, the concentrations of different N forms from non-mycorrhizal growing systems were similar with that of mycorrhizal growing systems (Figures 5, 6). All these results suggested that this mycorrhizal/non-mycorrhizal pair fully met the fourth criterion.

In summary, synthesizing results from our three experiments conducted in soils in the presence or absence of AM fungi, we did not observe any effects that could complicate interpretation of results related to the control of N loss control paddy fields via AM fungi. We conclude that this non-mycorrhizal/mycorrhizal rice pair represents a suitable combination for an additional, powerful experimental system for the scientific community examining the role of AM fungi in controlling N loss during rice production, and possibly other ecological questions related to rice. Further research is required in the future study on the performance of the two rice lines under other circumstances, such as during their entire life cycle, with other paddy soils and under lower levels of fertilization. Researchers interested in using this rice pair should conduct similar tests with their own soils to make sure that the use of this rice pair performs as expected.

## Data Availability Statement

All datasets generated for this study are included in the article/supplementary material.

## Author Contributions

SZ analyzed the data, wrote the first manuscript and modified it, designed the experiments, and applied funding to support the study. WY, YX, XG, and ZY performed the experiments, collected the samples, and collected the data. MR revised the manuscript and modified the language. All authors discussed the results and commented on the manuscript.

## Conflict of Interest

The authors declare that the research was conducted in the absence of any commercial or financial relationships that could be construed as a potential conflict of interest.
